# Inflammation mediates platinum-based chemotherapy-induced risk of adverse cardiac events in older NSCLC patients: a pilot study

**DOI:** 10.1186/s40959-026-00511-0

**Published:** 2026-05-20

**Authors:** Guoxing Zhang, Chao Liu, Xueying Zhang, Chunyi Jia, Baosheng Sun, Yongjie Wang, Jiming Xu, Linfeng Li, Haifeng Liu

**Affiliations:** 1 Department of Intensive Care Unit, Jilin Cancer Hospital, Changchun, 130000 China; 2 Yidu Cloud Technology Inc, Beijing, 100083 China; 3 Nanjing YiGenCloud Institute, Nanjing, 211899 China; 4 Medical Oncology, Jilin Cancer Hospital, Changchun, Changchun, 130000 China; 5 Department of Thoracic Surgery, Jilin Cancer Hospital, Changchun, 130000 China; 6 Department of Radiotherapy, Jilin Cancer Hospital, Changchun, 130000 China; 7 Cardio-oncology Department, Jilin Cancer Hospital, No. 1066 Jinhu Road, Changchun, Jilin 130000 China

**Keywords:** Non-small cell lung cancer, Chemotherapy, Causal mediation analysis, Adverse cardiac events

## Abstract

**Background:**

Chemotherapy increases the risk of cardiovascular toxicity, leading to adverse cardiac events (ACEs) in cancer patients. However, the underlying mechanisms and roles of mediators in this association remain poorly understood.

**Objectives:**

This study aimed to investigate the mediating role of inflammation in the association between platinum-based chemotherapy (PBC) and increased risk of ACEs in older patients with non-small cell lung cancer (NSCLC).

**Methods:**

We retrospectively analyzed data from NSCLC patients aged ≥ 50 years, initially diagnosed between 2018 and 2020 at our institution. The treatment group included patients who received PBC, whereas the control group consisted of patients who did not. The mediator was the maximum neutrophil-to-lymphocyte ratio (NLR) measured between initial chemotherapy and one year after diagnosis. The outcome was the occurrence of ACEs during follow-up period. Causal mediation analysis was conducted to assess the mediating effect of a clinically meaningful inflammatory response on the PBC–ACE association.

**Results:**

Among the 1,450 patients included (55% male, 45% female; median age: 61.6 years), 242 (16.7%) developed ACEs during the follow-up. The average direct effect was similar across both groups (control: 0.050, *p* = 0.018; treatment: 0.052, *p* = 0.018), indicating the consistent direct impact of PBC on the occurrence of ACE. However, the proportion of the total effect mediated by inflammation was higher in the treatment group (11.83%, *p* = 0.01) than that in the control group (9.12%, *p* = 0.01). Additionally, the average causal mediation effect was greater in the treatment group (0.0067, *p* < 0.01) than in the control group (0.0052, *p* < 0.001).

**Conclusions:**

Inflammation plays a crucial role in mediating the association between PBC and increased risk of ACEs in older patients with NSCLC. The mediating effect was more prominent in patients receiving PBC, emphasizing the need to monitor and manage inflammation to mitigate cardiovascular risks in patients with NSCLC undergoing PBC.

**Supplementary Information:**

The online version contains supplementary material available at 10.1186/s40959-026-00511-0.

## Background

Lung cancer is the most prevalent cancer worldwide, with non-small cell lung cancer (NSCLC) comprising most cases [1]. The global incidence of NSCLC continues to increase, with over 1 million new cases diagnosed annually [2]. Over the past few decades, survival rates have significantly improved owing to advances in treatment [3]. Consequently, cardiovascular disease has emerged as the leading cause of noncancer-related mortality in patients with NSCLC [4].

Platinum-based chemotherapy (PBC) plays a key role in the treatment of NSCLC but is well documented to be associated with adverse cardiac events (ACEs) [4, 5]. However, the underlying mechanisms remain poorly understood and warrant further investigation into the relationship between chemotherapy, its mechanisms, and cardiovascular complications [6].

Studies have identified several risk factors for adverse cardiac events, including inflammation and oxidative stress [7–9]. However, most of these studies have established associations, typically through correlation analysis or predictive modeling [10]. Few studies have applied causal inference methods—particularly quantitative approaches—to provide deeper insights. Compared with traditional association-based approaches, which identify correlations without establishing directionality, causal inference approaches aim to estimate the effect of an exposure under hypothetical scenarios. Despite their increasing use in other clinical fields, such methods have rarely been applied to understanding chemotherapy-related cardiotoxicity. This is yet to be achieved for several reasons. First, the sequence of events is often overlooked, which makes it difficult to establish causal relationships. Second, although emerging tools support causal inference using retrospective data, most are designed to estimate the overall causal effect of an exposure, and relatively few can investigate underlying mechanisms or pathways. Third, challenges related to data availability, such as insufficient biomarkers for inflammation or missing follow-up information, further complicate causal analyses in retrospective cohorts. Although standard inflammatory biomarkers such as C-reactive protein, IL-6, and TNF-α are commonly evaluated in cardio-oncology research, these markers were not routinely available in our retrospective dataset.

We, therefore, aimed to investigate the causal relationship among platinum-based chemotherapy (PBC), inflammation, and ACEs in older patients with NSCLC in a pilot proof-of-concept study. To address the challenges of establishing causality in retrospective data such as temporal sequencing, limited biomarkers, and missing follow-up information, we conducted a single-center retrospective preliminary analysis using a counterfactual framework. We selected the neutrophil-to-lymphocyte ratio (NLR) as a surrogate measure of systemic inflammation, as it is well validated, readily accessible from routine blood tests, and clinically meaningful in both oncologic and cardiovascular research. By applying advanced causal inference methods, we sought to fill the research gap and provide deeper insights into the mediating role of inflammation in PBC-associated cardiovascular risk.

## Methods

### Design and participants

**Fig. 1:**
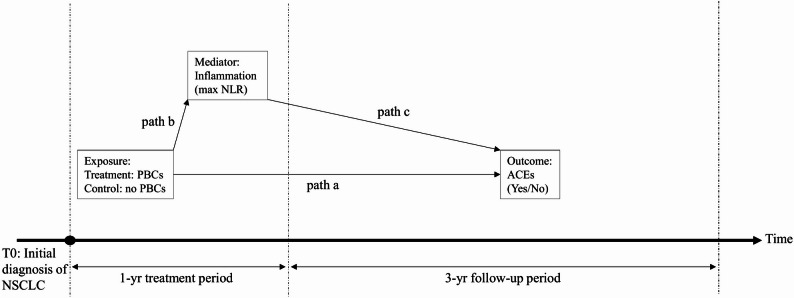
Structural causal diagram for the study. Path a displays the direct effect (DE) of treatment on ACEs risk, whereas paths b and c display the indirect effect (IE) mediated by inflammation. The sum of the DE and IE yields the total effect. NLR, Neutrophil-to-Lymphocyte Ratio; PBC, platinum-based chemotherapy; ACEs, adverse cardiac event; NSCLC, non-small cell lung cancer

This retrospective cohort study included patients aged ≥ 50 years who were newly diagnosed with histologically confirmed NSCLC of all stages between 2018 and 2020 at Jilin Cancer Hospital. The date of initial diagnosis (T0) was defined as the index time point. Patients were followed for a total of 4 years, comprising two contiguous and non-overlapping periods: a 0–1-year treatment period and a subsequent 1–4-year follow-up period (Fig. 1). Eligible patients had no documented history of cardiovascular events (e.g., coronary heart disease [CHD], myocardial infarction [MI]; see Table S2 for the full list) prior to T0 and received at least one documented systemic pharmacotherapy (chemotherapy, immunotherapy, or targeted therapy) within 1 year of diagnosis (referred to as the ‘treatment period’), ensuring that patients underwent active management. Patients without any recorded hospital visits during the follow-up period were excluded.

This study was conducted in accordance with the principles of the Declaration of Helsinki. This study was approved by the Ethics Board of Jilin Cancer Hospital (NO. 202504-001-01). The Institutional Review Board (IRB) of Jilin Cancer Hospital waived the requirement for informed consent because of the retrospective nature of the study.

### Exposure variables and mediators

The exposure of interest was the use of platinum-based chemotherapy (PBC; e.g., cisplatin, carboplatin) during the treatment period. The study cohort was first established based on the predefined inclusion and exclusion criteria, and all treatments were administered according to clinical needs under standard hospital protocols. Patients were then categorized as the ‘treatment group’ if they received any PBC-containing regimen, and as the ‘control group’ if their regimen did not include any PBC agents. Detailed information on drug treatments is provided in eTable 1 (Supplementary materials). Under a uniform care and management protocol, blood samples collected before treatment (for eligibility) and after treatment (for response assessment) provided all NLR measurements as part of routine clinical care. Inflammation, as reflected by the maximum neutrophil-to-lymphocyte ratio (NLR, calculated as the neutrophil count divided by the lymphocyte count) observed after the initial use of PBC (within the treatment period) in the treated group or the maximum NLR after initial therapy (regardless of type) in the control group, was used as the mediator [11, 12]. The NLR was treated as a continuous variable for the mediation analysis.

### Outcomes

The primary outcome was a composite of cardiovascular outcomes, defined as the occurrence of ACEs and selected related conditions during the follow-up period (hereafter collectively referred to as ACEs). Throughout this period, patients continued to receive routine clinical care, including regular re-evaluations based on clinical need and medical advice. Based on a retrospective review of the medical records, outcomes of interest included one or more of the following: (1) cardiac disease diagnosed using ICD codes (Table S2) and confirmed by ≥ 2 senior cardiologists; (2) pulmonary embolism or pulmonary artery embolism confirmed by CT imaging independently reviewed by two thoracic radiologists; (3) LVEF < 55% on echocardiography interpreted by qualified echocardiographers or cardiologists; or (4) elevated cardiac biomarkers, defined as cardiac troponin T (cTnT) > 50 ng/L or NT-proBNP > 450 ng/L in venous blood measured by the hospital’s certified clinical laboratory in accordance with National Center for Clinical Laboratory (NCCL) standards (2024). Patients who died during follow-up were treated as censored and classified as having no documented ACE prior to death.

### Covariates

The following covariates were initially included in the analysis due to their potential influence on treatment selection, mediators, and outcomes: (1) demographic factors: age at initial diagnosis and sex; (2) lifestyle factors: smoking status; (3) anthropometric measure: body mass index (BMI); (4) cancer stage; (5) medical history: diabetes, coronary heart disease, and hypertension; and (6) baseline NLR.

### Causal mediation analysis

To mitigate selection bias and preserve sample size, variables with excessive missing data were excluded. A complete case analysis was then performed. The hypothesized causal diagram investigated in this study is shown in Fig. 1. In this diagram, PBC was the independent variable, inflammation (as indicated by the maximum NLR after initial chemotherapy) served as the mediator, and the presence of ACEs was the outcome. The mediator was first regressed on exposure (path b), followed by modeling of the outcome variable with both chemotherapy status (platinum-based vs. others) and inflammation (paths a and c). All the retained covariates were included in the multivariate regression model.

Structural equation modeling was employed to jointly estimate these equations, accounting for within- and between-group variations in the datasets and the longitudinal nature of the data [13, 14]. A bias-corrected bootstrap analysis with 1,000 replications was conducted to estimate the average causal mediation effects, providing estimates for total, direct, and indirect (mediation) effects and their confidence intervals (CIs) without assuming normality [15].

The assumptions of causal mediation analysis must be evaluated to validate the analysis [16]. Specifically, we used a time-structured study design to address temporal precedence between the exposure, mediator, and outcome [17]. Furthermore, computational methods were employed to assess the no-interaction and no unmeasured confounding assumptions [18].

### Sensitivity analysis

Sensitivity analyses were conducted to evaluate the robustness of the results [16]. First, the Systemic Inflammation Response Index (SIRI), calculated as neutrophils* monocytes / lymphocytes, was used as the inflammatory mediator while all other model parameters and analytical procedures remained unchanged. Second, inflammation was redefined as a binary variable: “Well-controlled inflammation” required that the majority of a patient’s longitudinal NLR values (≥ 75%) remained within the normal range (< 3) and that no measurement reached the high-risk zone (maximum < 7) [19, 20]; “Not well-controlled inflammation” captured patients whose serial measurements showed either repeated elevations above normal or at least one episode of marked inflammation (NLR ≥ 7), indicating fluctuating or persistently inadequate inflammatory control [19, 21]. Third, variables initially excluded because of excessive missing data or due to concerns over collinearity and over-adjustment were reintroduced into the causal mediation analysis (CMA) as covariates, and a complete case analysis was conducted. The results were recorded despite the reduction in sample size as a consequence of the reintroduction of these variables. Finally, a CMA was performed using alternative outcome definitions (including a subset of the original ACEs).

### Statistical analysis

Normality of continuous variables was assessed using the Shapiro-Wilk test. Variables with normal distributions were compared using unpaired two-tailed t-tests, whereas non-normally distributed variables were analyzed using Wilcoxon rank-sum tests and reported as medians with interquartile ranges. Categorical variables were compared using chi-square tests. CMA was conducted using the ‘mediation’ R package, including the ‘medsens’ and ‘test.MINT’ functions, to evaluate the assumptions required for establishing causality. All statistical analyses were performed using R version 4.0.1 (R Foundation for Statistical Computing, Vienna, Austria). Statistical significance was set at p < 0.05.

## Results

### Patients

**Fig. 2:**
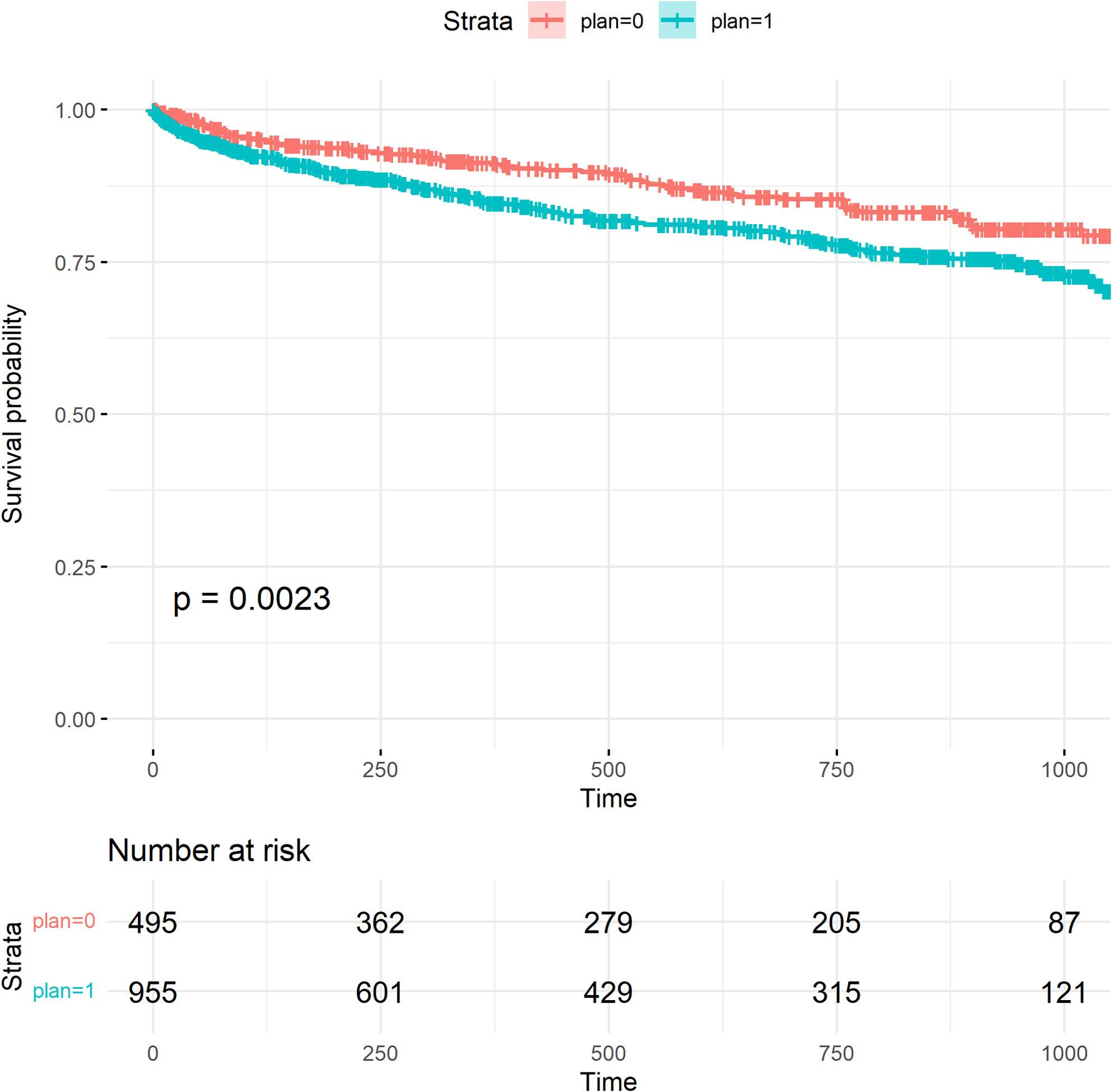
Survival curves (end event: ACEs) in different treatment groups. Treatment: patients receiving platinum-based chemotherapy (plan = 1) vs. controls: no platinum-based chemotherapy (plan = 0); end event: adverse cardiac events (ACEs). *Survival curves start a year after the initial diagnosis of NSCLC

A total of 1,450 patients (55% male, 45% female) were included in the retrospective observational cohort, with a median age at diagnosis of 61.6 years (IQR [56.2, 66.6]). Among patients with available staging information (*N* = 1,372), 27.3% were stage III, and 69.8% had advanced disease (stage III or IV). Overall, 955 patients (65.9% of the total cohort) received PBC during the treatment period (median number of cycles, 3 [IQR, 2–5]). Initiation of PBCs predominantly included cisplatin (56.1%) (Table S1). During follow-up, 242 (16.7%) patients developed ACEs and related events during follow-up (Table S2); the median interval between initial diagnosis and first presence of ACEs was 563.6 days (IQR, [420.7, 855.3]). A Kaplan–Meier curve was used descriptively to display survival curves for the treatment and control groups (Fig. 2).

A descriptive comparison of the variables between the treatment group (PBC group) and control group (no use of PBC) is presented in Table 1 (Additional variables that were not directly included in the modeling process, including detailed drug treatment information, are provided in eTable 1 in the Supplementary Materials). A higher proportion of male patients (587 out of 797, or approximately 73.7%) received PBC compared to female patients (368 out of 653, or approximately 56.4%); smoking status was also associated with the likelihood of receiving PBC, with smokers being more likely to receive the treatment (*p* < 0.01); patients receiving PBC were significantly younger, while the baseline NLR was significantly higher in patients receiving PBC compared to the control group; however, the absolute difference in both groups was small despite the statistical significance. Variables that may affect the use of PBC were included as covariates in the modeling process.

**Table 1:** Baseline demographic, clinical, and biological characteristics of patients who received platinum-based chemotherapy and those who did not

Characteristics	Treatment (PBCs) (*N* = 955)	Control (no PBCs) (*N* = 495)	*p*-value
Demographics			
Age	61.2 [56.0, 66.1]	62.4 [56.6, 67.3]	< 0.01
Sex			< 0.01
Female	368	285	
Male	587	210	
Lifestyle			
is_smoking*	326 (out of 575*)	140 (out of 345)	< 0.01
Illness history			
CHD	0	0	> 0.99
DM	103 (955)	63 (495)	0.31
HTH	200 (955)	115 (495)	0.35
Blood routine			
Baseline NLR	2.8 [1.9, 4.7]	2.6 [1.9, 3.6]	< 0.01
Anthropometry			
BMI*	23.3 [21.1, 25.4]	23.3 [21.2, 25.6]	0.75

Continuous variables were presented as medians (interquartile ranges), and categorical variables were presented as counts (percentages)

*CHD *Coronary heart disease, *DM* Diabetes mellitus, *HTN* Hypertension, *NLR* Neutrophil-to-lymphocyte ratio, *BMI* Body mass index

*Smoking status and BMI were missing; consequently, descriptive statistics were dependent on existing values

A descriptive comparison of baseline variables between the ACEs and non-ACE groups is presented in Table 2 (Additional variables that were not directly included in the modeling process are provided in eTable 2 in the Supplementary Materials). Age was positively associated with the risk of ACEs (*p* < 0.01), and no other baseline variables showed a statistically significant difference between patients who had ACEs and those who did not during follow-up.

**Table 2:** Baseline demographic, clinical, and biological characteristics of patients who presented adverse cardiac events (ACEs) and those who did not

Characteristics	ACEs (*N* = 242)	No ACEs (*N* = 1208)	*p*-value
Demographics			
Age	63.4 [58.2, 67.2]	61.4 [56.0, 66.3]	< 0.01
Sex			0.23
Female	100	553	
Male	142	655	
Lifestyle			
is_smoking*	86 (out of 155)	380 (out of 765)	0.22
Illness history			
CHD	0	0	> 0.99
DM	37 (242)	129 (1208)	0.05
HTN	56 (242)	259 (1208)	0.62
Blood routine			
Baseline NLR	2.78 [2.00, 3.93]	2.70 [1.91, 4.24]	0.61
Anthropometry			
BMI*	23.4 [20.9, 25.5]	23.3 [21.2, 25.4]	0.59

Continuous variables are presented as medians (interquartile ranges), and categorical variables are presented as counts (percentages)

*CHD* Coronary heart disease, *DM* Diabetes mellitus, *HTN* Hypertension, *NLR* Neutrophil-to-lymphocyte ratio, *BMI* Body mass index

*Smoking status and BMI were missing; consequently, descriptive statistics were dependent on existing values

### Mediators

The median number of NLR measurements obtained between the initiation of therapy-PBC for the treatment group and non-PBC systemic therapies for the control group (eTable 1)-and the end of the treatment period was 15 (interquartile range [IQR], 8–24), with group differences: treatment group median 19 (IQR: 11–28) vs. control group median 8 (IQR: 4–15). To illustrate the spatiotemporal patterns of post-initial therapy NLR dynamics, four patients were randomly selected for visualization, as presented in Figure S1.

### Casual mediation analysis

The results of the association between all covariates that passed missing-rate filtering and the maximum NLR (model 1 [path b]), as well as the association between the maximum NLR and the risk of ACEs (model 2 [path a and c]), are presented in Table S3. After adjusting for the retained covariates, a more severe inflammation status, as reflected by higher maximum NLR, was significantly associated with an increased risk of ACEs (OR = 1.019; 95% CI: 1.007–1.031).

Table 3 presents the results of the CMA results. The total effect, representing the combined direct and indirect effects of chemotherapy, was 0.05681 (95% CI: 0.01447–0.10, *p* = 0.01) across all participants. This total effect was significantly mediated by inflammation; however, there was a notable difference between the groups. The proportion mediated by inflammation was 0.09118 (95% CI: 0.02–0.38, *p* = 0.010) in the control group, and 0.1183 (95% CI: 0.02932–0.42, *p* = 0.01) in the treatment group. This indicated that a larger portion of the effect of chemotherapy on cardiac events was mediated by inflammation in the treatment group than in the control group.

**Table 3:** Summary of causal mediation analysis

	Estimate	95% CI Lower	95% CI Upper	*p*-value	Significance
ACME (control)	0.00518	0.00123	0.01	< 2e-16	***
ACME (treated)	0.00672	0.00170	0.02	< 2e-16	***
ADE (control)	0.05009	0.00840	0.09	0.018	*
ADE (treated)	0.05163	0.00875	0.09	0.018	*
Total Effect	0.05681	0.01447	0.10	0.010	**
Prop. Mediated (control)	0.09118	0.02040	0.38	0.010	**
Prop. Mediated (treated)	0.11825	0.02932	0.42	0.010	**
ACME (average)	0.00595	0.00147	0.01	< 2e-16	***
ADE (average)	0.05086	0.00863	0.09	0.018	*
Prop. Mediated (average)	0.10472	0.02492	0.40	0.010	**

*ACME* Average causal mediation effect, *ADE* Average direct effect

On average, across both groups, the proportion mediated was 0.1047 (95% CI: 0.02492–0.4, *p* = 0.01), further underscoring the differential mediation effect in the treated group. This suggests that inflammation plays a slightly more prominent role in mediating the chemotherapy–cardiac event relationship when patients receive treatment than when they do not. Our results highlight that inflammation significantly mediates the relationship between chemotherapy and ACEs, with a notably greater proportion of mediation observed in the treatment group than in the control group.

The assumption-checking results indicate that the mediation effect of the inflammatory response is sensitive to unmeasured confounding, whereas the direct effect is robust. No evidence of other violations of CMA assumptions was detected.

### Sensitivity analysis

We performed a sensitivity analysis to assess the robustness of our findings. First, variables initially excluded due to excessive missing data or concerns over collinearity and over adjustment were reintroduced as covariates in the causal mediation analysis. Second, a complete case analysis was conducted, including only patients whose smoking status or BMI data were not missing. While this greatly reduced the sample size, the average causal mediation effects and the trend of the proportion mediated in the treated group compared with the control group remained consistent, indicating that inflammation significantly mediated the association between PBC and increased ACE risk by approximately 10%, and the proportion of the total effect mediated by inflammation was higher in the treatment group. These results are detailed in the Supplemental Appendix (Table S4).

Next, when SIRI was used to reflect inflammation, the mediation results demonstrated patterns that were qualitatively and quantitatively consistent with the primary analysis (Table S4). Furthermore, under an alternative serial NLR-based definition of inflammation control as a binary mediator, 1,026 patients were classified as having not well-controlled inflammation and 424 as well-controlled inflammation. Survival analysis showed a significant difference in ACE-free survival between the two groups (*P* < 0.01; Figure S2). CMA using this mediator definition yielded results consistent with the primary analysis (Table S4).

Finally, we evaluated the sensitivity of our results by applying an alternative definition of the outcome. We strictly applied cardiac-specific events by excluding PE, and focused only on direct cardiac pathology. In such cases, the causal mediation effects remained significant and a higher proportion of mediation was observed in the treated group. All the quantitative results remained nearly unchanged (Table S4).

## Discussion

We applied CMA [22] and a counterfactual framework [23] to model the relationships between PBC, inflammation, and ACEs in patients with NSCLC. The results indicated that the contributing effects of PBC on ACEs were enhanced by inflammation (peak NLR) as well as by uncontrolled inflammation. This is the first study to apply the CMA framework to quantitatively analyze the relationship between cancer therapy, condition (inflammation), and cancer prognosis, facilitating the understanding of the magnitude and underlying mechanisms of adverse cardiovascular effects of platinum. Inflammation significantly mediated the association between PBC and an increased ACE risk by approximately 10%.

### Comparisons to prior studies

Our results are consistent with those of previous studies, which report a significantly increased risk of cardiovascular disease in testicular cancer survivors receiving cisplatin-based chemotherapy [24]. However, the pathway through which PBCs increase the risk of ACEs has not yet been elucidated. From a methodological perspective, most existing observational studies apply methods such as propensity score matching or Mendelian randomization to estimate the overall causal effect of an exposure. In contrast, CMA allows the decomposition of effects into direct and indirect pathways, enabling investigation of the mechanisms linking PBC to adverse cardiac events. The application of CMA in this study therefore offers an important complement to the existing literature by shedding light on the potential mediating role of inflammation. Supporting this mechanistic hypothesis, a prior study revealed that the association between cancer and coronary artery disease (CAD) was significant among patients with high inflammation rather than among patients with low inflammation, based on regression analysis of two groups of patients with different inflammatory statuses (NLR-high vs. NLR-low) [6]. Extending these findings, we used CMA to provide evidence that PBC increases the risk of ACEs through inflammation. In addition to confirming the elevated risk associated with PBC and the role of inflammation, our results provide evidence based on an integrated framework that incorporates the three (i.e., PBC, inflammation, and ACEs) while considering their sequential order, and consequently, the potential causality of inflammation.

### Inflammation in cardiotoxicity

Inflammation plays an important role in cardiotoxicity [25]. These underlying mechanisms are multifaceted and interconnected. Firstly, platinum compounds, such as cisplatin, carboplatin, and oxaliplatin, induce oxidative stress and reactive oxygen species (ROS) production [26], which can lead to cellular damage and subsequent activation of inflammatory pathways [27, 28]. This oxidative stress can trigger the release of pro-inflammatory cytokines, such as TNF-α, IL-1β, and IL-6, which are central to the inflammatory response [24, 29]. These cytokines can disrupt endothelial function, leading to vascular dysfunction and an increased risk of thromboembolic events, thereby exacerbating the cardiovascular burden [30–32]. Secondly, inflammation can directly impair cardiac function by promoting cardiomyocyte apoptosis and fibrosis, as evidenced by studies showing an increased expression of inflammatory markers and cardiac injury biomarkers in patients undergoing anti-cancer chemotherapy [33]. Activation of the nuclear factor kappa-light-chain-enhancer of activated B cells (NF-κB) and other inflammatory signaling pathways further amplify the inflammatory cascade, contributing to endothelial hyperpermeability and cardiac tissue damage [30, 34]. Additionally, inflammation can modulate the gut microbiota, as demonstrated in animal models where alterations in the microbial composition are associated with enhanced cardiotoxicity [35, 36]. Dysbiosis may further propagate systemic inflammation and create a vicious cycle of inflammation and cardiac injury [37]. Collectively, these mechanisms provide a plausible explanation for the mediating role of inflammation in the association between PBC and ACEs, highlighting the importance of targeting inflammation as a potential strategy to mitigate cardiotoxicity.

Several inflammatory biomarkers, including C-reactive protein, IL-6, and TNF-α, have been used in related studies [25]. In the present study, we used the NLR as a measure of systemic inflammation, as it is well validated, readily available from routine complete blood counts, and clinically informative in both oncology and cardiovascular medicine. Biologically, NLR reflects the balance between neutrophil-driven pro-inflammatory activity and lymphocyte-mediated immune regulation, making it a practical proxy for systemic inflammation. Prior studies have consistently linked NLR with cancer prognosis, cardiovascular risk, and atherosclerotic burden, supporting its relevance for evaluating an inflammation-mediated pathway [38, 39]. Nonetheless, NLR may fluctuate over time, particularly during acute illness, and a single measurement may not fully capture chronic inflammatory status. Additionally, inconsistent sampling during the treatment period represents another limitation. Although sampling was systematically performed before and after treatment, the intervals between measurements varied, which may have introduced imprecision in estimating the true inflammatory burden. To mitigate these limitations, we selected the peak NLR within a predefined time window (detailed in the Methods-Exposure Variables and Mediators) to reflect a clinically meaningful inflammatory response. We also conducted sensitivity analyses leveraging repeated measures of NLR (detailed in the Methods-Sensitivity Analysis).

### Clinical implications

Considering the significant role of inflammation in mediating the association between PBCs and ACEs, several clinical implications can help mitigate adverse outcomes. First, controlling inflammation before initiating chemotherapy is crucial because pre-existing inflammatory conditions may enhance the connection between PBCs and ACEs. This can be achieved using anti-inflammatory medications, lifestyle modifications, or underlying inflammatory diseases [40]. Second, monitoring inflammation status during and after PBC is essential. The regular assessment of inflammatory biomarkers such as C-reactive protein and interleukin-6 (IL-6) can provide early indications of inflammation and allow timely interventions to prevent or mitigate cardiac complications [41, 42]. Finally, for patients undergoing PBC, especially those with (pre-existing) inflammation, regular screening for ACEs is paramount. This includes frequent echocardiographic evaluations, cardiac biomarker monitoring, and advanced imaging techniques such as cardiac magnetic resonance to detect subclinical changes in cardiac function [43]. By implementing these strategies, clinicians can proactively manage inflammation and its downstream effects on cardiac health, ultimately improving patient outcomes and reducing the chemotherapy-induced cardiotoxicity (CIC) burden.

### Limitations

As a pilot and proof-of-concept investigation, this study provides valuable insights into the causal mediating effects of inflammation on the association between PBC and ACEs. However, this study had some limitations. First, it was conducted at a single center, which may have introduced a selection bias and limited the generalizability of the findings. The potential causal relationships identified here need to be validated in multicenter studies to account for diverse patient populations and clinical practices. Second, the retrospective design limits the ability to draw definitive causal conclusions; therefore, the effects should be interpreted partially as associational [44]. Although CMA provides insight into potential mechanisms, future research incorporating longitudinal or prospective designs would enable more robust assessment and better control of confounding. In addition, results should be interpreted within the study’s methodological framework, including the predefined time frame, follow-up duration, end-event definitions, and the selection of analytical parameters and markers. Third, unmeasured confounding represents a limitation inherent to causal mediation analysis using retrospective data [45]. Specifically, granular treatment-level details—such as drug dosages, concurrent medications, and other therapeutic interventions—could not be accurately extracted from the EHR and were therefore not included, which may affect the precision of our estimates.

### Strengths of the study

Despite the limitations, this pilot study is the first to elucidate the causal mediating effects of inflammation in the context of PBC and ACEs. To mitigate potential weaknesses, we ensured a large and adequate sample size to increase statistical power, thereby enhancing the reliability of the findings. Additionally, we adopted stringent inclusion criteria to minimize the influence of pre-therapy conditions (and their associated indices/markers) on the results, ensuring that the observed effects were more likely attributable to the treatment itself and the mediator. Finally, we conduct sensitivity analyses to validate the robustness of our findings, providing further confidence in the conclusions drawn. Future research should build on these initial findings through multicenter prospective studies with comprehensive control of confounders to fully elucidate the role of inflammation in chemotherapy-induced cardiotoxicity.

## Conclusions

This retrospective pilot study utilizes a CMA framework to elucidate the mediating effects of inflammation between PBC and ACEs in older patients with NSCLC. Our findings highlight the significant role of inflammation in this association, suggesting that controlling inflammation is crucial to mitigate the effects of PBC on ACEs.

## Supplementary Information

Supplementary Material 1.

## Data Availability

The datasets used and/or analyzed in the current study are available from the corresponding author upon reasonable request.

## Abbreviations

ACE: Adverse cardiac event
CMA: Causal mediation analysis
NLR: Neutrophil-to-lymphocyte ratio
NSCLC: Non-small cell lung cancer
PBC: Platinum-based chemotherapy
