# Supplementary material for: Inflammation mediates platinum-based chemotherapy-induced risk of adverse cardiac events in older NSCLC patients: a pilot study
Source: Cardiooncology. 2026 May 20;12:92. doi: 10.1186/s40959-026-00511-0 (PMC13360043; doi:10.1186/s40959-026-00511-0)
Supplement: Supplementary file 1 — Supplementary Material 1. [file 40959_2026_511_MOESM1_ESM.docx]

**Supplemental Materials**

**Figures**

**Figure S1**. The NLR spatiotemporal pattern representation for four randomly-selected patients from our study cohort.


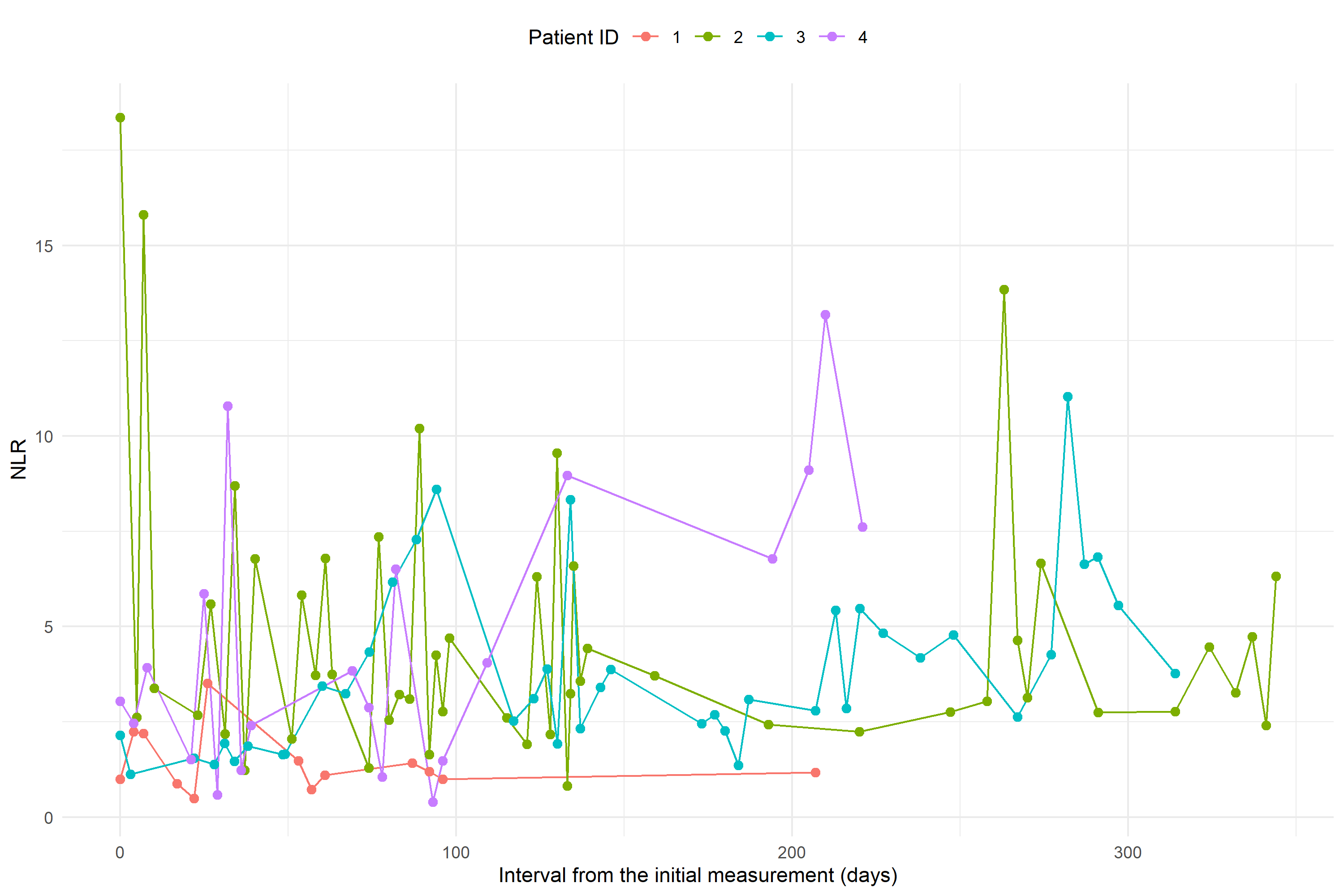


NLR: neutrophil to lymphocyte ratio.

Notes: Patient IDs were de-identified and converted to numerics (i.e., 1, 2, 3, 4).

**Figure S2**. Descriptive comparison of survival curves (end event: adverse cardiac events [ACEs]) in groups with different inflammation status.


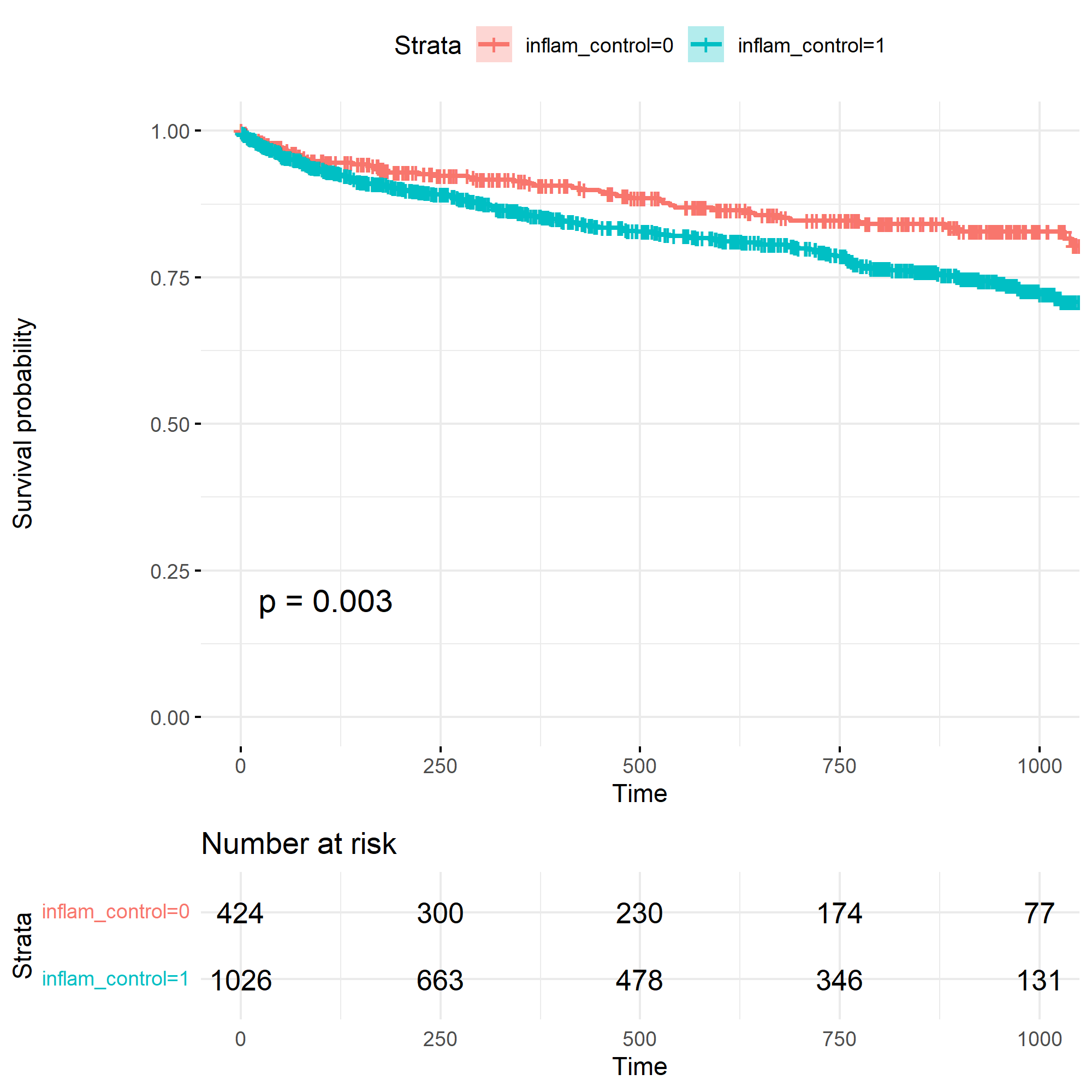


Inflam_control = 0 (well-controlled inflammation) vs. inflam_control = 1 (poorly controlled inflammation). End event: adverse cardiac events (ACEs).

*Survival curves start 1 year after the initial diagnosis of NSCLC.

**Tables**

**Table S1**. Distribution of the initial platinum-based compounds in the treatment group.

| **NO** | **Drug Name** | **Frequency** |
| --- | --- | --- |
| 1 | Oxaliplatin Mannitol | 5 |
| 2 | Oxaliplatin | 15 |
| 3 | Cisplatin | 536 |
| 4 | Carboplatin | 185 |
| 5 | Lobaplatin | 89 |
| 6 | Nedaplatin | 125 |
| 7 | total | 955 |

Note: frequency represents the first-used type of platinum-based compound for each patient in the treatment group.

**eTable 1**. Extended version of Table 1 with additional supplementary information.

| **Characteristics** | **Treatment (PBCs)**  **(N = 955)** | **Control (no PBCs)**  **(N = 495)** | **p-value** |
| --- | --- | --- | --- |
| **Histology** |  |  | <0.01 |
| Adenocarcinoma | 526 | 416 |  |
| Squamous cell carcinoma | 353 | 57 |  |
| Adenosquamous carcinoma | 11 | 4 |  |
| Large cell carcinoma | 14 | 0 |  |
| Unspecified/unavailable | 51 | 18 |  |
|  |  |  |  |
| **Stage** |  |  | <0.01 |
| I | 138 | 106 |  |
| II | 150 | 20 |  |
| III | 313 | 62 |  |
| IV | 306 | 277 |  |
| Unspecified/Unavailable | 48 | 30 |  |
|  |  |  |  |
| **Mutational status** |  |  |  |
| BRAF (15)  Mutation positive (4)  Mutation negative (11) | 7  3  4 | 8  1  7 | 0.46 |
| MET (15)  Mutation positive (4)  Mutation negative (10)  p.P488L (exon 4, missense) (1) | 5  2  3  0 | 10  2  7  1 | 0.59 |
| KRAS (25)  Mutation positive (17)  Mutation negative (8) | 14  13  1 | 11  4  7 | 0.01 |
| RET (10)  Mutation positive (2)  Mutation negative (6)  Fusion (2) | 4  1  3  0 | 6  1  3  2 | 0.43 |
| ROS1 (54)  Mutation positive (10)  Mutation negative (44) | 34  6  28 | 20  4  16 | >0.99 |
| ALK (79)  Mutation positive (23)  Mutation negative (56) | 43  11  32 | 36  12  24 | 0.61 |
| EGFR (289)  Mutation positive (213)  Mutation negative (74)  Exon 20 insertion (2) | 161  103  56  2 | 128  110  18  0 | <0.01 |
|  |  |  |  |
| **Treatment** |  |  |  |
| **Surgery** |  |  |  |
| Yes | 282 | 122 | 0.057 (yes no) |
| Lobectomy | 229 | 88 | <0.01 |
| Wedge resection | 58 | 35 |  |
| Segmentectomy | 8 | 12 |  |
| No/unavailable | 673 | 373 |  |
|  |  |  |  |
| **Radiotherapy** |  |  |  |
| Yes | 311 | 87 | <0.01 |
| Site: Brain | 83 | 37 | <0.01 |
| Lung | 177 | 28 |  |
| Bone | 31 | 21 |  |
| Neck | 8 | 3 |  |
| Gland | 4 | 0 |  |
| Others | 286 | 73 |  |
| No/unavailable | 644 | 408 |  |
| **Radiotherapy + other treatment regimens** * |  |  | - |
| Radiotherapy + chemotherapy | 20 | 2 |  |
| Radiotherapy + targeted therapy | 7 | 9 |  |
| Radiotherapy + targeted therapy + immunotherapy + chemotherapy | 1 | 0 |  |
| Radiotherapy + immunotherapy | 4 | 1 |  |
| Radiotherapy + immunotherapy + chemotherapy | 2 | 0 |  |
| Radiotherapy + targeted therapy + chemotherapy | 0 | 1 |  |
|  |  |  |  |
| **Chemotherapy** |  |  |  |
| Yes | 955 | 83 | <0.01 |
| No/unavailable | 0 | 412 |  |
|  |  |  |  |
| **Targeted therapy** |  |  |  |
| Yes | 269 | 200 | <0.01 |
| No/unavailable | 686 | 295 |  |
|  |  |  |  |
| **Immunotherapy** |  |  |  |
| Yes | 110 | 10 | <0.01 |
| No/unavailable | 845 | 485 |  |
|  |  |  |  |
| **Other medications**^#^ |  |  |  |
| Glucocorticoids | 867 | 250 | <0.01 |
| NSAID | 272 | 83 | <0.01 |
| Immunosuppressants | 148 | 28 | <0.01 |
| immune checkpoint inhibitors | 67 | 10 | <0.01 |

NSAID: nonsteroidal anti-inflammatory drugs

Notes: Numbers in parentheses following each gene name indicate the number of patients with available mutational testing results.

*Data are presented for patients with non-missing information on combined treatment regimens only.

#Drug use information between initial diagnosis and peak NLR occurrence.

**eTable 1** (continued)

| **Category** | **Subcategory** | **Drugs**  **(Chinese)** | **Drugs**  **(English)** | **Treatment (PBCs)**  **(N = 955)** | **Control (no PBCs)**  **(N = 495)** |
| --- | --- | --- | --- | --- | --- |
| **Targeted Therapy**  (15) | EGFR Tyrosine Kinase Inhibitors (EGFR-TKIs) | 吉非替尼 (1^st^ gen) 埃克替尼 厄洛替尼 阿法替尼 (2^nd^ gen) 奥希替尼 (3^rd^ gen) | Gefitinib Icotinib Erlotinib Afatinib Osimertinib | √  √  √  √  √ | √  √  √  √  √ |
|  | ALK Tyrosine Kinase Inhibitors (ALK-TKIs) | 克唑替尼 (1^st^ gen) 阿来替尼 (2^nd^ gen) 塞瑞替尼 | Crizotinib Alectinib Ceritinib | √  √  √ | √  √  √ |
|  | Other TKIs | 伊马替尼 依维莫司 | Imatinib Everolimus | √ | √ |
|  | Anti-EGFR mAb | 西妥昔单抗 | Cetuximab | √ |  |
|  | Anti-HER2 mAb | 曲妥珠单抗 帕妥珠单抗 | Trastuzumab Pertuzumab | √ | √  √ |
|  | Antiangiogenic | 贝伐珠单抗  重组人血管内皮抑制素 | Bevacizumab  Recombinant human endostatin (Endostar) | √  √ | √  √ |
| **Immunotherapy**  (5) | PD-1 inhibitors | 帕博利珠单抗 卡瑞利珠单抗 信迪利单抗 纳武利尤单抗 特瑞普利单抗 | Pembrolizumab Camrelizumab Sintilimab Nivolumab Toripalimab | √  √  √  √  √ | √  √  √  √ |
| **Chemotherapy**  (24) | Platinum-based | 顺铂 卡铂 奈达铂 洛铂 奥沙利铂 奥沙利铂甘露醇 | Cisplatin Carboplatin Nedaplatin Lobaplatin Oxaliplatin Oxaliplatin mannitol | √  √  √  √  √  √ |  |
|  | Taxanes | 紫杉醇 多西他赛 | Paclitaxel Docetaxel | √  √ | √  √ |
|  | Antimetabolites | 培美曲塞二钠 吉西他滨 卡培他滨 替吉奥 氟尿嘧啶 雷替曲塞 | Pemetrexed Gemcitabine Capecitabine Tegafur (S-1) 5-Fluorouracil Raltitrexed | √  √  √  √  √  √ | √  √  √  √  √  √ |
|  | Topoisomerase inhibitors | 伊立替康 依托泊苷 | Irinotecan Etoposide | √  √ | √  √ |
|  | Vinca alkaloids | 长春瑞滨 | Vinorelbine | √ | √ |
|  | Alkylating agents | 替莫唑胺 环磷酰胺 异环磷酰胺 | Temozolomide Cyclophosphamide Ifosfamide | √  √ | √  √  √ |
|  | Anthracyclines | 吡柔比星 表柔比星 多柔比星脂质体 博来霉素 | Pirarubicin Epirubicin Liposomal doxorubicin Bleomycin | √  √  √ | √  √  √ |
| **Supportive Care**  (3) | Cytokines | 重组人白介素-11 重组人白介素-2 | Recombinant human IL-11 Recombinant human IL-2 | √  √ | √  √ |
|  | COX-2 inhibitors | 塞来昔布 | Celecoxib | √ | √ |
| **Other**  (1) | Traditional medicine | 榄香烯 | Elemene | √ | √ |

Notes: Numbers in parentheses denote the number of unique drug entities (by name) within each treatment category.

**eTable 2**. Extended version of Table 2 with additional supplementary information.

| **Characteristics** | **ACEs**  **(N = 242)** | **No ACEs**  **(N = 1208)** | **p-value** |
| --- | --- | --- | --- |
| **Histology** |  |  | 0.42 |
| Adenocarcinoma | 145 | 797 |  |
| Squamous cell carcinoma | 80 | 330 |  |
| Adenosquamous carcinoma | 2 | 13 |  |
| Large cell carcinoma | 2 | 12 |  |
| Unspecified/unavailable | 13 | 56 |  |
|  |  |  |  |
| **Stage** |  |  | 0.031 |
| I | 29 | 215 |  |
| II | 23 | 147 |  |
| III | 72 | 303 |  |
| IV | 111 | 472 |  |
| Unspecified/Unavailable | 7 | 71 |  |
|  |  |  |  |
| **Mutational status** |  |  |  |
| BRAF (15)  Mutation positive (4)  Mutation negative (11) | 2  1  1 | 13  3  10 | >0.99 |
| MET (15)  Mutation positive (4)  Mutation negative (10)  p.P488L (exon 4, missense) (1) | 2  1  1  0 | 13  3  9  1 | 0.70 |
| KRAS (25)  Mutation positive (17)  Mutation negative (8) | 5  5  0 | 20  12  8 | 0.24 |
| RET (10)  Mutation positive (2)  Mutation negative (6)  Fusion (2) | 2  1  1  0 | 8  1  5  2 | 0.43 |
| ROS1 (54)  Mutation positive (10)  Mutation negative (44) | 8  1  7 | 46  9  37 | >0.99 |
| ALK (79)  Mutation positive (23)  Mutation negative (56) | 13  4  9 | 66  19  47 | >0.99 |
| EGFR (289)  Mutation positive (213)  Mutation negative (74)  Exon 20 insertion (2) | 45  31  13  1 | 244  182  61  1 | 0.33 |
|  |  |  |  |
| **Treatment** |  |  |  |
| **surgery** |  |  |  |
| Yes | 46 | 358 | <0.01 |
| Lobectomy | 39 | 278 | 0.52 |
| Wedge resection | 10 | 83 |  |
| Segmentectomy | 4 | 16 |  |
| No/unavailable | 196 | 850 |  |
|  |  |  |  |
| **Radiotherapy** |  |  |  |
| Yes | 96 | 302 | <0.01 |
| Site: Brain | 23 | 97 | 0.59 |
| Lung | 55 | 150 |  |
| Bone | 12 | 40 |  |
| Neck | 4 | 7 |  |
| Gland | 1 | 3 |  |
| Others | 81 | 278 |  |
| No/unavailable | 146 | 906 |  |
| **Radiotherapy + other treatment regimens** * |  |  |  |
| Radiotherapy + chemotherapy | 4 | 18 |  |
| Radiotherapy + targeted therapy | 3 | 13 |  |
| Radiotherapy + targeted therapy + immunotherapy + chemotherapy | 0 | 1 |  |
| Radiotherapy + immunotherapy | 1 | 4 |  |
| Radiotherapy + immunotherapy + chemotherapy | 0 | 2 |  |
| Radiotherapy + targeted therapy + chemotherapy | 0 | 1 |  |
|  |  |  |  |
| **Chemotherapy** |  |  |  |
| Yes | 190 | 848 | 0.01 |
| No/unavailable | 52 | 360 |  |
|  |  |  |  |
| **Targeted therapy** |  |  |  |
| Yes | 97 | 372 | <0.01 |
| No/unavailable | 145 | 836 |  |
|  |  |  |  |
| **Immunotherapy** |  |  |  |
| Yes | 33 | 87 | <0.01 |
| No/unavailable | 209 | 1121 |  |
|  |  |  |  |
| **Other medications** |  |  |  |
| Glucocorticoids | 194 | 923 | 0.24 |
| NSAID | 44 | 311 | 0.02 |
| Immunosuppressants | 45 | 131 | <0.01 |
| immune checkpoint inhibitors | 22 | 55 | 0.01 |

NSAID: nonsteroidal anti-inflammatory drugs

Notes: Numbers in parentheses following each gene name indicate the number of patients with available mutational testing results.

*Data are presented for patients with non-missing information on combined treatment regimens only.

#Drug use information between initial diagnosis and peak NLR occurrence.

**Table S2**. List of adverse cardiac events.

| **NO** | **Chinese Name** | **English Name** | **ICD-10 Code** |
| --- | --- | --- | --- |
| 1 | 冠状动脉粥样硬化 | Coronary Artery Atherosclerosis | I25.1 |
| 2 | 冠心病 | Coronary Heart Disease (CHD) | I25.9 |
| 3 | 缺血性心肌病 | Ischemic Cardiomyopathy | I25.5 |
| 4 | 冠状动脉性心脏病 | Coronary Artery Heart Disease | I25.9 |
| 5 | 缺血性心肌病 | Ischemic Cardiomyopathy | I25.5 |
| 6 | 心房颤动 | Atrial Fibrillation (AF) | I48.0 |
| 7 | 心绞痛 | Angina Pectoris | I20.9 |
| 8 | 慢性心功能不全 | Chronic Heart Failure (CHF) | I50.9 |
| 9 | 心脏病 | Heart Disease | I25.9 |
| 10 | 心梗 | Myocardial Infarction (MI) | I21.9 |
| 11 | 心房恶性肿瘤 | Malignant Tumor of the Atria | C32.0* |
| 12 | 心肌梗塞 | Myocardial Infarction (MI) | I21.9 |
| 13 | 心肌缺血 | Myocardial Ischemia | I25.2 |
| 14 | 心脏支架 | Cardiac Stent (Coronary Artery Stent) | Z95.5 |

*Atrial tumors, such as atrial myxomas, may be coded under this

**Table S3**. Sub-models of the causal mediation analysis

|  | **Dependent variable:** | |
| --- | --- | --- |
|  | **Max NLR** | **ACEs (0/1)** |
|  | **OLS**  **(model 1)** | **Logistic**  **(model 2)** |
| **Max NLR** |  | 0.019***  (0.006) |
| **plan** | 2.445***  (0.614) | 0.392**  (0.163) |
| **age** | 0.024  (0.040) | 0.031***  (0.010) |
| **sex** | 0.413  (0.578) | 0.062  (0.148) |
| **Baseline NLR** | 0.062  (0.073) | -0.022  (0.021) |
| **dm** | 0.308  (0.902) | 0.371*  (0.208) |
| **htn** | -0.496  (0.697) | 0.024  (0.174) |
| **Constant** | 7.260***  (2.569) | -4.034***  (0.667) |
| **Observations** | 1,450 | 1,450 |
| **R²** | 0.014 |  |
| **Adjusted R²** | 0.010 |  |
| **Log Likelihood** |  | -638.408 |
| **Akaike Inf. Crit.** |  | 1,292.815 |
| **Residual Std. Error** | 10.719 (df = 1443) |  |
| **F Statistic** | 3.484*** (df = 6; 1443) |  |

**Significance Levels**:* p < 0.1, ** p < 0.05, *** p < 0.01

The standard errors are provided in parentheses below each coefficient.

Abbreviations: NLR, neutrophil-to-lymphocyte ratio; DM, diabetes mellitus; htn, hypertension

Note: chd is zero across all patients and was thus omitted from the modeling process.

Plan: 1, platinum-based chemotherapy; 0, no platinum-based chemotherapy

Model 1: Association between all covariates (***X***) and maximum NLR (y)

Model 2: Association between all covariates, maximum NLR (***X***), and risk of ACEs (y)

**Table S4**. Sensitivity analysis results

|  | BMI Added  (N = 1188) | | Smoking Added  (N = 920) | | Smoking + BMI (Simultaneous Inclusion) (N = 920) | | Well-Controlled vs. Not  (N = 1450) | | Alternative Outcome Definition  (N = 1450) | |
| --- | --- | --- | --- | --- | --- | --- | --- | --- | --- | --- |
|  | **Estimate** | **p-value** | **Estimate** | **p-value** | **Estimate** | **p-value** | **Estimate** | **p-value** | **Estimate** | **p-value** |
| **ACME (control)** | 0.004917  (0.000800, 0.01) | 0.010 ** | 0.00441  (0.000065, 0.01) | 0.044 * | 0.00443  (0.000061, 0.01) | 0.044 * | 0.004869 (0.000353, 0.01) | 0.030 * | 0.00515 (0.00120, 0.01) | <2e-16 *** |
| **ACME (treated)** | 0.005996  (0.000923, 0.01) | 0.010 ** | 0.00532  (0.000071, 0.02) | 0.044 * | 0.00535  (0.000071, 0.02) | 0.044 * | 0.006312 (0.000400, 0.01) | 0.030 * | 0.00666 (0.00167, 0.02) | <2e-16 *** |
| **ADE (control)** | 0.038515  (-0.005313, 0.08) | 0.094 . | 0.0377  (-0.016, 0.08) | 0.166 | 0.0377  (-0.016, 0.08) | 0.166 | 0.049230 (0.009506, 0.09) | 0.018 * | 0.04946 (0.00791, 0.09) | 0.02 * |
| **ADE (treated)** | 0.039594  (-0.005590, 0.08) | 0.094 . | 0.0386  (-0.0166, 0.09) | 0.166 | 0.0386  (-0.0164, 0.09) | 0.166 | 0.050673 (0.009676, 0.09) | 0.018 * | 0.05097 (0.00816, 0.09) | 0.02 * |
| **Total Effect** | 0.044510  (-0.000660, 0.09) | 0.052 . | 0.043  (-0.00811, 0.09) | 0.106 | 0.043  (-0.0084, 0.09) | 0.106 | 0.055542 (0.013405, 0.10) | 0.010 ** | 0.05612 (0.01402, 0.10) | 0.01 ** |
| **Prop. Mediated (control)** | 0.110462  (-0.107411, 0.80) | 0.062 . | 0.102  (-0.508, 1.13) | 0.150 | 0.103  (-0.515, 1.07) | 0.146 | 0.087660 (0.003862, 0.34) | 0.040 * | 0.09177 (0.02042, 0.39) | 0.01 ** |
| **Prop. Mediated (treated)** | 0.134702  (-0.091885, 0.81) | 0.062 . | 0.124  (-0.461, 1.13) | 0.150 | 0.124  (-0.475, 1.07) | 0.146 | 0.113638 (0.005284, 0.37) | 0.040 * | 0.11875 (0.02933, 0.42) | 0.01 ** |
| **ACME (average)** | 0.005456  (0.000877, 0.01) | 0.010 ** | 0.00486  (0.000068, 0.01) | 0.044 * | 0.00489  (0.000071, 0.01) | 0.044 * | 0.005590 (0.000376, 0.01) | 0.030 * | 0.00591 (0.00144, 0.01) | <2e-16 *** |
| **ADE (average)** | 0.039054  (-0.005451, 0.08) | 0.094 . | 0.0382  (-0.0162, 0.08) | 0.166 | 0.0382  (-0.0161, 0.08) | 0.166 | 0.049951 (0.009544, 0.09) | 0.018 * | 0.05022 (0.00804, 0.09) | 0.02 * |
| **Prop. Mediated (average)** | 0.122582  (-0.099648, 0.80) | 0.062 . | 0.113  (-0.484, 1.13) | 0.150 | 0.114  (-0.495, 1.07) | 0.146 | 0.100649 (0.004573, 0.35) | 0.040 * | 0.10526 (0.02492, 0.41) | 0.01 ** |

Signif. codes: 0 ‘***’ 0.001 ‘**’ 0.01 ‘*’ 0.05 ‘.’ 0.1 ‘ ’ 1

Notes: Alternative definition/subset of outcomes (strictly cardiac-specific events): Exclude pulmonary embolism (PE) and focus only on direct cardiac pathology.

**Table S4**. Sensitivity analysis results (continued)

|  | SIRI as mediator  (N = 1457) | | TNM stage and histology added  (N = 1308) | | Concurrent medications added  (N = 1450) | |
| --- | --- | --- | --- | --- | --- | --- |
|  | **Estimate** | **p-value** | **Estimate** | **p-value** | **Estimate** | **p-value** |
| **ACME (control)** | 0.00430  (0.00113, 0.01) | 0.004 ** | 0.003083  (−0.000286, 0.01) | 0.094 . | 0.004052  (0.000588, 0.01) | 0.012 * |
| **ACME (treated)** | 0.00583  (0.00157, 0.01) | 0.004 ** | 0.003915  (−0.000398, 0.01) | 0.094 . | 0.004993  (0.000754, 0.01) | 0.012 * |
| **ADE (control)** | 0.05720  (0.01892, 0.10) | 0.004 ** | 0.048105  (0.005073, 0.09) | 0.046 * | 0.041252  (−0.001923, 0.08) | 0.068 . |
| **ADE (treated)** | 0.05873  (0.01950, 0.10) | 0.004 ** | 0.048938  (0.005303, 0.09) | 0.046 * | 0.042193  (−0.001952, 0.08) | 0.068 . |
| **Total Effect** | 0.06304  (0.02447, 0.10) | <2e-16 *** | 0.052020  (0.009642, 0.10) | 0.036 * | 0.046245  (0.002731, 0.09) | 0.042 * |
| **Prop. Mediated (control)** | 0.06824  (0.01658, 0.23) | 0.004 ** | 0.059261  (−0.019816, 0.39) | 0.126 | 0.087612  (−0.004662, 0.53) | 0.054 . |
| **Prop. Mediated (treated)** | 0.09253  (0.02472, 0.27) | 0.004 ** | 0.075261  (−0.025921, 0.42) | 0.126 | 0.107964  (−0.005437, 0.55) | 0.054 . |
| **ACME (average)** | 0.00507  (0.00136, 0.01) | 0.004 ** | 0.003499  (−0.000342, 0.01) | 0.094 . | 0.004522  (0.000692, 0.01) | 0.012 * |
| **ADE (average)** | 0.05797  (0.01926, 0.10) | 0.004 ** | 0.048521  (0.005188, 0.09) | 0.046 * | 0.041723  (−0.001938, 0.08) | 0.068 . |
| **Prop. Mediated (average)** | 0.08039  (0.02166, 0.25) | 0.004 ** | 0.067261  (−0.023081, 0.41) | 0.126 | 0.097788  (−0.005050, 0.54) | 0.054 . |

Signif. codes: 0 ‘***’ 0.001 ‘**’ 0.01 ‘*’ 0.05 ‘.’ 0.1 ‘ ’ 1

Notes: SIRI: Systemic Inflammation Response Index; Concurrent medications: NSAID, immunosuppressants, and immune checkpoint inhibitors.
